# Research Progress on the Preparation of Manganese Dioxide Nanomaterials and Their Electrochemical Applications

**DOI:** 10.3390/nano14151283

**Published:** 2024-07-30

**Authors:** Chunsheng Xie, Zesheng Xu, Yujian Zheng, Shuo Wang, Min Dai, Chun Xiao

**Affiliations:** 1 College of Environmental and Chemical Engineering, Zhaoqing University, Zhaoqing 526061, China; xiechsh@126.com (C.X.); xuzesheng0712@163.com (Z.X.); zhengyujian10032@163.com (Y.Z.); daimin1007@163.com (M.D.); 2 Guangdong Provincial Key Laboratory of Environmental Health and Land Resource, Zhaoqing University, Zhaoqing 526061, China; 3 School of Environmental and Chemical Engineering, Xi’an Polytechnic University, Xi’an 710048, China; 220621098@stu.xpu.edu.cn; 4 State Environmental Protection Key Laboratory of Water Environmental Simulation and Pollution Control, South China Institute of Environmental Sciences, Ministry of Ecology and Environment, Guangzhou 510655, China

**Keywords:** nano-MnO_2_, preparation method, structure, electrochemical applications

## Abstract

Manganese dioxide (MnO_2_) nanomaterials have shown excellent performance in catalytic degradation and other fields because of their low density and great specific surface area, as well as their tunable chemical characteristics. However, the methods used to synthesize MnO_2_ nanomaterials greatly affect their structures and properties. Therefore, the present work systematically illustrates common synthetic routes and their advantages and disadvantages, as well as examining research progress relating to electrochemical applications. In contrast to previous reviews, this review summarizes approaches for preparing MnO_2_ nanoparticles and describes their respective merits, demerits, and limitations. The aim is to help readers better select appropriate preparation methods for MnO_2_ nanomaterials and translate research results into practical applications. Finally, we also point out that despite the significant progress that has been made in the development of MnO_2_ nanomaterials for electrochemical applications, the related research remains in the early stages, and the focus of future research should be placed on the development of green synthesis methods, as well as the composition and modification of MnO_2_ nanoparticles with other materials.

## 1. Introduction

MnO_2_ nanomaterials stand out among other nanomaterials owing to their good environmental compatibility, low cost, and strong oxidative and adsorptive properties. Owing to their good biocompatibility, optical physical properties, and chemical properties [1,2], these nanomaterials are used as catalysts [3] and in electrochemistry [4], biomedicine [5], and materials sciences [6], among other fields. MnO_2_ nanomaterials have different spatial structures and therefore have different crystalline forms, mainly α-MnO_2_, β-MnO_2_, γ-MnO_2_, δ-MnO_2_, and λ-MnO_2_. The surface physicochemical characteristics of MnO_2_ vary considerably based on the crystal structure. Based on their spatial structure, MnO_2_ nanomaterials can be categorized into having a one-dimensional (1D) tunnel structure, a two-dimensional (2D) layered structure, or a three-dimensional (3D) network structure [7].

**Figure 1:**
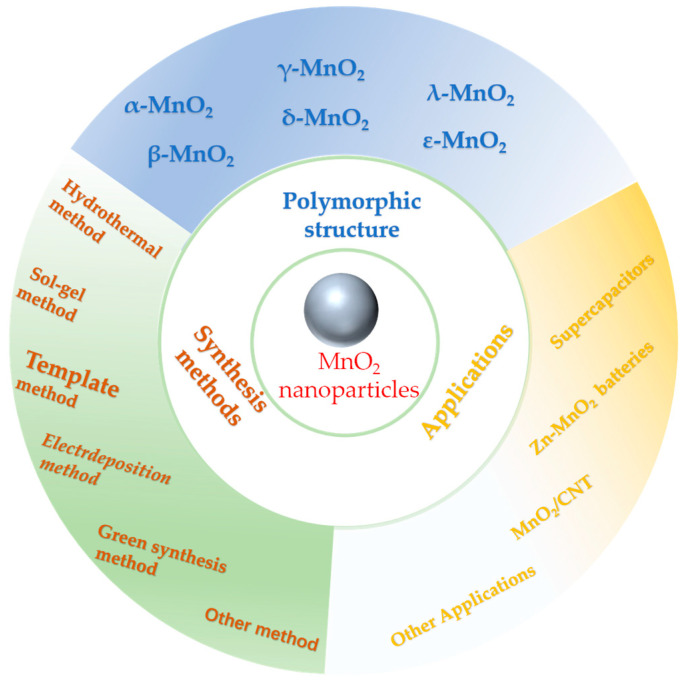
Preparation methods and applications of MnO_2_ nanoparticles and polymorphic structures.

The synthesis method of MnO_2_ nanomaterials crucially impacts their electrochemical performance. It has been shown that chemical synthesis methods yield MnO_2_ nanomaterials with poor electrochemical performance, such as low capacity attenuation and low cycling efficiency, due to side reactions and defects in synthesis. Contrarily, hydrothermal methods can yield MnO_2_ nanomaterials with crystal structures that are favorable for charge transfer and ion diffusion. Therefore, they usually exhibit enhanced electrochemical performance. The electrochemical deposition method enables MnO_2_ to be directly deposited on the electrode; therefore, it has high controllability and a long cycle life, as well as conferring enhanced electrochemical performance. By studying the synthesis of MnO_2_ nanomaterials, their applications in electrochemistry have been substantially improved. However, there is an enormous gap between theory and practice. Therefore, this review summarizes the research progress on MnO_2_ nanomaterials in recent years, with the aim of helping readers to better select preparation methods for MnO_2_ nanomaterials and translate research results into practical applications, as shown in Figure 1. Compared with previous reviews [8,9], this review contains the latest research results in this field in recent years, and these advances have not only improved the performance and stability of MnO_2_ nanomaterials but can also be applied across several fields. Recent research has placed much focus on preparing MnO_2_ nanomaterials using the green synthesis method, a synthetic method that has been studied more and more in recent years, and which indicates that greening preparation methods will be an important direction for the future.

## 2. Structure of MnO_2_ Nanoparticles

The polymorphic phases of MnO_2_ usually comprise crystalline and amorphous phases. The crystalline phase comprises octahedral units; they can form either layered or chain/tunnel structures when different joining methods are used [10,11,12]. The interconnection of MnO_6_ octahedra forms c-axis paralleling chains within the crystal structure, along with tunnels between these chains. The different polymorphs can be associated with Mn^4+^ arrangement, as each polymorph contains a hexagonal close-packed lattice structure composed of O^2−^ and Mn^4+^ [13].

The structures of different manganese dioxide materials are shown in Table 1. α-MnO_2_, β-MnO_2_, and γ-MnO_2_ have 1D (1 × 1)/(2 × 2), (1 × 1)/(1 × 1), and (1 × 1)/(1 × 2) tunneling structures, respectively. However, β-MnO_2_ has a smaller tunneling structure, which is unfavorable for rapid ion transport, while α-MnO_2_ has a larger tunneling structure, which is favorable for ion embedding and detachment. ε-MnO_2_ has an alike structure to γ-MnO_2_; however, the manganese lattice sites are arranged in a disorderly manner, with irregular tunneling. δ-MnO_2_ has a 2D laminar structure formed on the MnO_6_ octahedra side; this structure facilitates rapid ion transport with low preparation cost and high specific surface area. And λ-MnO_2_ has the representative spinel structure with the 3D (1 × 1) tunnel structure; this structure excels in electrochemical performance. The varying atomic configurations within these different crystalline phases result in a diverse array of pores, which have implications on the electrolyte ion migration or electron transfer processes within the charge storage mechanisms.

## 3. Synthesis of MnO_2_ Nanomaterials

MnO_2_ nanomaterials have been extensively studied as environmentally friendly catalysts. Their preparation methods include the hydrothermal method [20], sol–gel [21], template [22], electrochemical method [23], and coprecipitation [24] methods. Each of these methods has different degrees of effects on the particle size distribution, grain size, and crystal transformation of MnO_2_ nanomaterials. Moreover, the properties, structure, and morphology of MnO_2_ nanomaterials are considerably influenced by the synthesis conditions. To synthesize MnO_2_ nanomaterials with specific structures, morphologies, and sizes for practical production or experiments, studying the synthesis methods and conditions is vital. Nine commonly used methods for synthesizing manganese dioxide are described below, all of which have unique advantages, potential drawbacks, and a wide range of applications.

### 3.1. Hydrothermal Method

The hydrothermal method involves synthesizing materials via chemical reactions in water under high temperature and pressure using the water solubility of inorganic compounds. MnO_2_ nanomaterials with different morphologies can be obtained by changing temperature and pressure [25].

**Figure 2:**
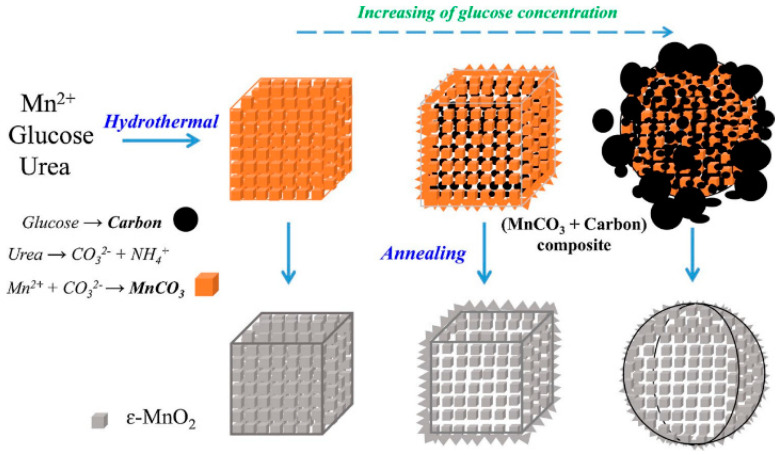
Schematic diagram of porous ε-MnO_2_ microcubes [30].

Chen et al. [26] prepared β-MnO_2_, γ-MnO_2_, and δ-MnO_2_ using the hydrothermal approach and α-MnO_2_ via solid-phase synthesis, and investigated their catalytic properties for the oxidation of benzene and formaldehyde. The results showed that α-MnO_2_ and γ-MnO_2_ outperformed δ-MnO_2_ and β-MnO_2_ in benzene oxidation, while δ-MnO_2_ was more active in formaldehyde oxidation. Oxygen was found to exert the catalytic effect on oxidizing formaldehyde and benzene, as elucidated through the quantitative correlation between specific oxygen content and reaction rate. Yang et al. [27] prepared α-MnO_2_ solid and hollow sea urchins via hydrothermal synthesis. The 3D α-MnO_2_ hollow sea urchin was analyzed for the post-plasma toluene catalytic decomposition. The carbon dioxide selectivity, toluene decomposition, and carbon balance of α-MnO_2_ hollow sea urchin were ~59%, ~100%, and ~81%, respectively, which were 96%, 43%, and 44% higher than the non-thermal plasma process. These values were also higher than those for the α-MnO_2_ solid sea urchin. Aljafari et al. [28] used α-MnO_2_ and Cu-MnO_2_ nanoparticles as candidate materials for counter electrode materials (CEs) and synthesized them with the simple hydrothermal approach under 140 °C and 14 h. Among those prepared Dye-Sensitized Solar Cell (DSSCs), the 10 wt% Cu-doped MnO_2_ cathode showed the highest energy conversion efficiency of 1.7%, whereas the Power Conversion Efficiency (PCE) of pristine MnO_2_ was only 1.21%. The results indicated that Cu-MnO_2_ nanoparticles exhibited superior electrocatalytic ability for DSSCs than α-MnO_2_. Table 2 summarizes the environmental applications of MnO_2_ prepared by the hydrothermal method. Clearly, MnO_2_ has good applications in heavy metal adsorption, organic pollutant adsorption, and catalysts. Especially, Figure 2 illustrates the preparation process of porous ε-MnO_2_ with the assistance of the solvent, MnO_2_ showed high porosity and the best performance of the catalyst preparation at the 6-2-6 (ε-MnO_2_ of Mn-6-2-6) manganese glucose-urea ratio. Therefore, it is necessary to pay attention to the molar ratio of solvent in the hydrothermal synthesis of MnO_2_.

**Table 2:** Hydrothermal preparation of MnO_2_ and applications.

Structure of MnO_2_	Targets	Synthesis Conditions	Results	Applications	Reference
δ-MnO_2_	Pb (II) and U (VI)	-	The adsorption capacities were 41.32 and 492.61 mg g^−1^, respectively	Adsorbent	[29]
Pristine ε-MnO_2_ and ε-MnO_2_ of Mn-6-2-6	Toluene	Manganese (II) nitrate hexahydrate, urea, glucose 180 °C	The conversion 41% and 85%, respectively	Catalysts	[30]
MnO_2_	Tl (I)	KMnO_4_, MnSO_4_·H_2_O, 240 °C	Adsorption capacity was 450 mg g^−1^	For removing thallium (Tl) from wastewater	[31]
MnO_2_ nanoparticles	MB (Methylene Blue)	KMnO_4_, CH_3_CH_2_OH, HCl	The adsorption capacities 22.2 mg g^−1^ after 60 min.	Removal of MB	[32]
α-MnO_2_, β-MnO_2_, and δ-MnO_2_	MG (Methyl Glucoside)	-	The removal efficiency of MG 96.42%, 46.58%, 99.75%, respectively	For typical organic pollutant removal	[33]
MnO_2_ nanostructures	-	KMnO_4_, Mn (CH_3_COO)_2_	The capacitance was 348.2 F g^−1^ and rate capability of 89% for 2000 cycles.	Electrode materials	[34]
δ-MnO_2_	-	Mn-MOF, KMnO_4_, 120 °C	The capacitance was 416 F g^−1^	Capacitors	[35]

In summary, hydrothermal synthesis is an economical and excellent method, and it has the following advantages. (1) It can yield high-purity products and (2) the as-synthesized products have excellent properties. (3) It enables us to precisely adjust nanoparticle size and morphology, and the final nanoparticle size and morphology are influenced by changing reaction system pH value, and (4) no organic solvent is needed by the synthesis reagents. However, its primary drawbacks lie in the requirement for costly equipment and stringent reaction conditions during synthesis, coupled with relatively lengthy reaction periods [36].

### 3.2. Sol–Gel Approach

In the sol–gel approach, homogeneous solutions are formed within the solvent using metal-alcohol salts or inorganic salts as precursors. The solute interacts with the solvent or other solutes via hydrolysis and condensation. The solution is condensed into a gel, which is then heated and subjected to later treatments to obtain the eventual target product. Different temperatures, pH, solution concentrations, and reaction duration may affect the reaction system and yield products with different phases [37]. Although the sol–gel method is less studied for synthesizing MnO_2_, it has numerous possible merits compared with traditional synthetic approaches. For instance, it is often used to synthesize optical and photovoltaic hybrid materials [38].

**Figure 3:**
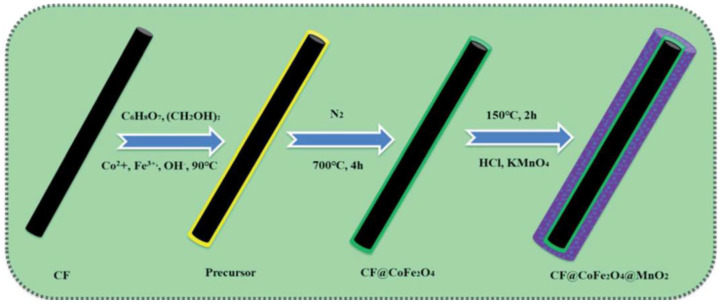
Process for the preparation of CF@CoFe_2_O_4_@MnO_2_ composites [46].

Reddy et al. [39] synthesized MnO_2_ with a sol–gel approach and by reducing aqueous NaMnO_4_ using an organic reducing agent, i.e., fumaric acid. The difference in pore size distribution between the two materials resulted in two forms of manifold. Compared with the dry gel, the manifold exhibited higher capacitance. MnO_2_ has a higher capacitance in two moles of sodium chloride electrolyte than other electrolytes like potassium chloride, sodium sulfate, and lithium chloride. According to previous research [40], MnO_2_ nanoparticles and Ag-doped MnO_2_ nanoparticles were prepared using the sol–gel approach. The decomposition of methyl orange (MO) and phenol via MnO_2_ was evaluated upon visible light irradiation. The results showed that the MnO_2_ catalyst doped with a Ag volume fraction of 10 exhibited higher photocatalytic efficiency for MO than phenol. In addition, Ag-doped MnO_2_ catalysts can be used for wastewater treatment and for removing environmental contaminants. Kusworo et al. [41] prepared a photocatalyst composite (ZnO-MnO_2_@SiO_2_) using the sol–gel approach, and later prepared the polysulfone/ZnO-MnO_2_@SiO_2_ (PSf/ZnO-MnO_2_@SiO_2_) membrane through the non-solvent-induced phase separation technique. Incorporating the ZnO-MnO_2_@SiO_2_ photocatalyst could enhance membrane hydrophilicity, porosity, mechanical strength and water absorption capacity. Moreover, the recyclability, flux stability, and antifouling performances of the membrane improved under UV light irradiation, thereby preventing scale formation and prolonging the membrane life span. Thus, the PSf/ZnO-MnO_2_@SiO_2_ membrane was used for natural rubber-containing wastewater treatment. Table 3 summarizes the electrochemical applications of MnO_2_ nanomaterials prepared by the sol–gel method, from which it is known that MnO_2_ nanomaterials has good applications in supercapacitors. Figure 3 illustrates the preparation process of carbon fiber @cobaltferrite@manganese dioxide (CF@CoFe_2_O_4_@MnO_2_) composites by sol–gel method and hydrothermal reaction. Notably, the CF@CoFe_2_O_4_@MnO_2_ nanomaterials can also have good magnetic behavior in microwave absorbers.

**Table 3:** Structure and application of MnO_2_ nanomaterials prepared using the sol–gel method.

MnO_2_ Structure	Synthesis Conditions	Result	Applications	Reference
γ-MnO_2_	MnAc_2_·4H_2_O, C_6_H_8_O_7_·H_2_O	Capacitance was 317 F g^−1^	Supercapacitors	[42]
Mesoporous Silica/MnO_2_ composite (MS/MnO_2_)	Tetraethyl Orthosilicate, KMnO_4_	Capacitance was 1158.50 F g^−1^	Supercapacitors	[43]
Nanostructured MnO_2_	-	The capacitance was 627.9 F g^−1^	Supercapacitors	[44]
Nickel-doped layered MnO_2_	KMnO_4_, Ni (NO_3_)_2_·6H_2_O	The capacitance was 140 mAh g^−1^	Sodium-ion batteries	[45]
CF@CoFe_2_O_4_@MnO_2_	FeCl_3_·6H_2_O, CoCl_2_·6H_2_O, CF (Carbon Fiber), KMnO_4_	The microwave absorbing capacity can reach up −41 dB	Microwave absorbers	[46]
α-MnO_2_ and Cu-α-MnO_2_	CuSO_4_·5H_2_O, KMnO_4_	The maximum degradation of Methylene Blue (MB) by α-MnO_2_, 1% Cu-α-MnO_2_, 5% Cu-α-MnO_2_, and 10% Cu-αMnO_2_ were 97.9%, 98.3%, 98.7%, and 99.5%, respectively	Degradable MB	[47]

In summary, the sol–gel approach can be a simple technique for controlling the reaction at a molecular level, which yields products with fine, high-purity, homogeneous morphologies and crystal structure [48]. Using the sol–gel method, a thin protective coating can be fabricated to ensure good adhesion between the substrate and the top layer. However, the method has drawbacks like long synthesis time and complex operation steps.

### 3.3. Template Method

In recent years, the template method is commonly used for nanomaterial synthesis using various structure-directing agents or templates. Using organic molecules as template agents, guest species and surfactant molecules are co-assembled to the regular microstructure via template guidance to generate self-assembled nanomaterials with ordered structures. Template methods can be categorized into soft and hard template methods according to the used template type. The entire process is broadly divided into three steps: (1) template synthesis, (2) MnO_2_ synthesis according to the template, and (3) template deletion or retention in line with the requirements [49].

#### 3.3.1. Soft Template Approach

The soft template approach typically utilizes nonrigid nanostructures as the templates, which are generated through intermolecular interactions. Subsequently, inorganic source deposition onto nonrigid soft template interior and surface yields mesostructures with well-defined dimensions and pore structures. Surfactants, flexible organic molecules, and block copolymers are generally used as soft templates for interacting with metal ions and merging to liquid crystal phases using the sol–gel method. The mesostructures with open pores were acquired when the soft template was removed via calcination. Depending on concentration of surfactants, micelles of different morphologies are formed. These micellar structures allowed inorganic materials to exhibit specific distribution trends driven by electrostatic interactions between surfactant molecules and nanomaterials, hydrogen bonding, and van der Waals forces [50]. Hou et al. [51] found that micelles are an important factor in controlling shape synthesis. However, soft templates can be adjusted to produce various MnO_2_ nanomaterials by adjusting precursor concentrations and reaction conditions [52].

Yuan et al. [53] used polymers as soft templates for directing MnO_2_ nanowire growth and stabilizing their structure to form the special graphene-loaded MnO_2_ nanowires. The nanostructures exhibited excellent catalytic activity for oxidizing organic pollutants in neutral and alkali environments. They demonstrated that the morphology of MnO_2_ considerably influenced the catalytic performance of MnO_2_. Tran et al. [54] synthesized mesoporous MnO_2_ nanoparticles by olefinic oxidation using permanganate within a soft template solution. Asymmetric capacitors, with activated carbon and MnO_2_ as the cathode and anode separately, were assembled and investigated in aqueous potassium sulfate solution. Experimental results showed that mesoporous MnO_2_ nanoparticles were the candidate electrode material used in electrochemical energy storage because of their superb low-power capacitive performance. Yang et al. [55] developed the in situ soft template reduction method for the deposition of exposed and well-dispersed MnO_2_ nanoparticles in mesoscopic channels within the regular ordered mesoporous Ce-based metal-organic framework (OMUiO-66(Ce)). The substrate channel promoted hydrogen peroxide decomposition with MnO_2_ as the catalyst; it also exhibited great efficiency, persistent intracellular antioxidant effects and low-dose activity. The developed MnO_2_@OMUiO-66(Ce) had considerable potential for application and could efficiently reduce the oxidative stress.

The soft template approach has numerous merits. For instance, soft templates are available in various forms, and can be prepared by an easy and economical way, with no need of complex instrument. Indeed, soft templates also possess certain drawbacks such as imprecise control over size and shape, difficulties in template removal, challenges in achieving high product purity, and potential contamination from byproducts. These limitations need to be carefully considered when utilizing soft templates for various applications.

#### 3.3.2. Hard Template Approach

The above-mentioned soft template approach has limitations such as uneasy control of product size, morphology or uniformity. Moreover, the remaining macromolecules, organic compounds, and surfactants probably enhance ionic resistivity [56]. On the contrary, the hard template method effectively decreases interference as no surfactant is involved. Compared with the soft template approach, the hard template approach shows a promising application in synthesizing MnO_2_ nanostructures.

**Figure 4:**
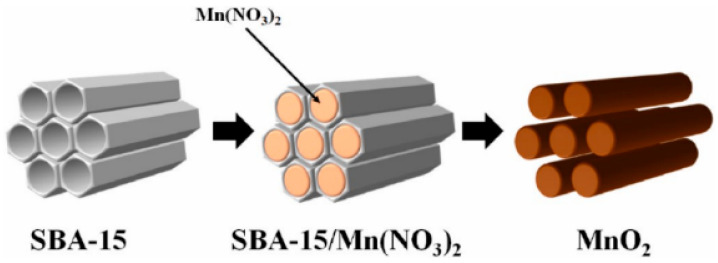
Schematic showing the mesoporous MnO_2_ preparation through nano-casting with the ordered mesoporous SBA-15 material being the hard template [59].

Bai et al. [57] used KIT-6 to be the hard template to synthesize a 3D regular mesoporous MnO_2_ (3D-MnO_2_). 3D-MnO_2_ had large specific surface area, templated mesoporous properties, and cubic symmetry. 3D-MnO_2_ made it possible for formaldehyde to completely convert into water and carbon dioxide. The excellent catalytic activity of 3D-MnO_2_ might be associated with the great specific surface area, special mesoporous structure, and numerous surfaces Mn^4+^ ions. Zhang et al. [58] synthesized mesoporous MnO_2_ (M-MnO_2_) via nano-casting by using porous silica SBA-15 as a hard template. M-MnO_2_ exhibited an 8-fold increased adsorption capacity for phenol compared with control MnO_2_ (C-MnO_2_). Hydroxyl radicals were identified as major reactive oxygen species, while the concentration of hydroxyl radical from M-MnO_2_ was increased by about two times compared with that from C-MnO_2_. Zhang et al. [59] also synthesized M-MnO_2_ catalysts with increased pore size, pore volume, and specific surface area The oxidation reactivity of M-MnO_2_ for oxalic acid (OA) and MO was evaluated. The results showed that the M-MnO_2_ catalysts were most potent for catalyzing MO and OA degradation, with degradation efficiencies of 98.37% and 92.96%, respectively. Figure 4 displays the above MnO_2_ synthetic process. Table 4 summarizes the environmental applications of MnO_2_ synthesized using the hard template approach, MnO_2_ has good application properties in supercapacitors, batteries, catalysts, etc.

Nonetheless, template utilization leads to the higher cost of synthesis. Wang et al. [64] prepared graded MnO_2_, in which cotton and potassium permanganate were the template and precursor, respectively. Compared to additional templates, the biomaterial is environmentally-friendly and easily available, and cotton fibers have homogeneous morphology compared with other plant fibers. From an economic point of view, the environmentally friendly, cost-effective, and sustainable bio-template approach is applicable to synthesizing MnO_2_ nanomaterials.

Generally, the hard template method has the following advantages compared with other synthesis methods: (1) the template can be used as a carrier for synthesizing nanomaterials of various shapes, (2) it solves the problem of the dispersion stability of nanomaterials, with the realization of the synthesis and assembly of the integration, and (3) the operation process is simple and suitable for mass production [65]. However, there are drawbacks like the high cost of the templates and the contamination from byproducts.

### 3.4. Electrodeposition Method

Electrodeposition is commonly used to prepare thin films and nanoparticles [66]. The deposit morphology, physicochemical properties and crystal structure are adjusted by changing the electrodeposition conditions, such as voltage, current [67], deposition time [68], and electrolyte concentrations [69]. Therefore, the electrochemical method is advantageous relative to others, and its properties include that it is (1) controllable, (2) simple and easy to operate, (3) has a relatively low processing temperature, and (4) has mild reaction conditions. MnO_2_ electrodeposition proceeds as follows:

Mn^2+^ + 2H_2_O → MnO_2_ + 2e^−^ + 4H^+^.

**Figure 5:**
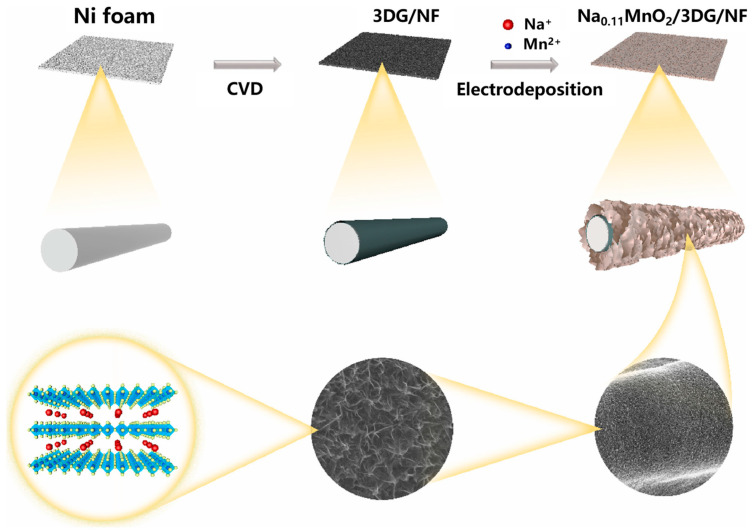
Schematic of the synthesis process of Na_0.11_MnO_2_/3DG composites [71].

The deposition potential and conditions considerably affect the oxidation state, structure, surface area, and properties of MnO_2_ [70]. Ren et al. [71] used the easy electrodeposition approach to prepare Na^+^ pre-intercalated δ-MnO_2_ nanosheets (Na_0.11_MnO_2_) onto 3D graphene (3DG). The specific capacitance of Na_0.11_MnO_2_/3DG electrodes was 1240 F g^−1^ at the 0.2 A g^−1^ current density. Moreover, Na_0.11_MnO_2_/3DG showed high cycle stability, and the capacitance retention of the electrolyte was 90% following 9000 cycles within 2 mol ZnSO_4_/0.2 mol or MnSO_4_ aqueous solution. The above study provided a new perspective for δ-MnO_2_ to be a cathode with excellent energy and power density for energy-storage devices. The Na_0.11_MnO_2_/3DG material preparation process is shown in Figure 5. Shi et al. [72] deposited reduced nickel (rNi) bases via secondary construction on nanocore nickel foam materials. These bases had a great specific surface area and improved active substance mass utilization. The electrodeposition of MnO_2_ on reduced nickel bases could be achieved via pre-intercalation treatment using Na^+^, K^+^, and NH_4_^+^ three cations. Moreover, the mechanism of diverse monovalent cations guiding MnO_2_ material growth was analyzed. The rNi/MnO_2_ composite with the unique nano-sintered structure could be acquired via electrodeposition on reduced nickel bases. Supercapacitors assembled using this electrode exhibited extremely high special capacitance as well as energy densities of 80.22 and 24.90 W kg^−1^ at the 599.99 and 11,997.98 W kg^−1^ power densities, respectively. Zhao et al. [73] used ultrathin nanosheets to prepare MnS_2_/MnO_2_-Carbon Cloth (MnS_2_/MnO_2_-CC) heterostructure bifunctional catalysts via the two-step electrodeposition approach for MB degradation in organic wastewater. These catalysts required overpotentials as low as 66 and 116 mV for achieving 10 and 100 mA cm^−2^ current densities within the MB/H_2_SO_4_ medium. They also had superb stability (with performance retention during 24-h testing) and a low Tafel slope (26.72 mV dec^−1^). The MB degradation rate reached 97.76%, which is considerably increased relative to the 72.10% rate of the MnO_X_-CC catalyst. The study provided a novel idea for synthesizing stable and high-efficiency nonprecious metal bifunctional electrocatalysts to conduct out HER and degradation of organic wastewater. Table 5 summarizes the environmental applications of MnO_2_ synthesized through electrodeposition, MnO_2_ can be used in supercapacitors, catalysts, and batteries.

In summary, the performance of MnO_2_ nanomaterials prepared via electrochemical deposition does not have high electrical conductivity, resistance, and specific capacitance compared with those synthesized using other methods [82]. However, nanocomposites such as carbon nanorods synthesized using this method have promising applications.

### 3.5. Reflux Approach

The reflux method is the wet chemical preparation approach, which requires no high-temperature calcination. The reflux method can synthesize nanomaterials with the same particle size and excellent catalytic performance directly. This approach is advantageous in the simple operation, mild reaction conditions, and excellent purity of synthesized materials [83]. Moreover, it can be used in large-scale MnO_2_ nanoparticle synthesis.

Zhang et al. [84] used the simple microwave-assisted reflux method without using templates and surfactants to synthesize γ-MnO_2_ and α-MnO_2_ nanoparticles via 5 min refluxing in neutral and acidic environments separately. Similarly, single-crystal β-MnO_2_ nanorods (length, 0.5~2 µm; diameter, 20~50 nm) were prepared via reflux treatment with potassium permanganate and manganese (II) sulfate within a nitric acid solution. In addition, the reflux method is applicable for synthesizing doped nanoparticles. Said et al. [85] converted γ-MnO_2_ into ε-MnO_2_ morphology by controlling the reflux reaction temperature and time. The TGA/DTA results showed γ-MnO_2_ had higher stability compared with ε-MnO_2_. Moreover, reaction temperature considerably impacted the product phase and surface properties according to surface area analysis. The thermal behavior and magnetic properties of MnO_2_ were also investigated. May et al. [86] synthesized α-MnO_2_ via refluxing using nitric oxide and investigated how two synthesis methods affected the catalytic activities of CuO/α-MnO_2_ catalysts. The relations of catalytic CO oxidation capacity with structural properties were explored. The results showed that CO and abundant surface oxygen could be found at the catalyst’s interfacial sites, inferring that the catalytic performance of the CuO/MnO_2_ catalyst depended on CO adsorption onto the reduced copper oxide.

However, the reflux method has some drawbacks, and the quality of the as-obtained product is influenced by several factors. Kijima et al. [87] prepared α-, β-, and γ-MnO_2_ with three phase structures by acid digestion using MnO_2_ trioxide under repetitive conditions. MnO_2_ products had a polymorphic type, which was tightly associated with reaction temperature as well as the acid type and concentration. α-MnO_2_ was formed by reaction at high sulfuric acid concentrations and low temperatures. On the contrary, β-MnO_2_ could be acquired by reaction under low sulfuric acid concentrations and high temperatures. γ-MnO_2_ was obtained under intermediate conditions between β-MnO_2_ and α-MnO_2_. Only β-MnO_2_ and γ-MnO_2_ were synthesized using nitric acid, whereas β-MnO_2_ was formed under harsher conditions compared to γ-MnO_2_, with higher temperatures and higher nitric acid concentrations.

In summary, the reflux method for preparing MnO_2_ nanomaterials boasts advantages such as simplicity of operation, mild reaction conditions, and high purity of the synthesized material. Furthermore, it is suitable for large-scale synthesis of MnO_2_ nanoparticles. However, the quality of the obtained MnO_2_ nanomaterials was influenced by a multitude of factors.

### 3.6. Microemulsion Approach

Microemulsions are clear liquid phases (monophases) with high thermodynamic stability formed from water, oil, surfactants, and co-surfactants. Water and oil are immiscible, and surfactants are amphiphilic. Different from common emulsions, microemulsions can be generated after blending water, oil and surfactants without the requirement of high-shear conditions. Direct (oil dispersed within water), reverse (water dispersed in oil), and bi-continuous and supercritical carbon dioxide are four microemulsion types. The microemulsion method is used to synthesize well-controlled, narrow, monodispersed nanoparticles [88]. It is mainly used to homogeneously synthesize metal nanoparticles (diameters, 5–50 nm) [89]. This method demonstrates high practicability and efficiency in synthesizing and processing inorganic nanomaterials, which is beneficial for uniform volume heating, energy saving and higher reaction rate than conventional heating methods.

Xu et al. [90] synthesized MnO_2_ with a particle diameter of ~4 nm by the microemulsion method. Compared to chemical coprecipitation, the particle size of MnO_2_ considerably decreased. The capacitance value of MnO_2_ was 246.2 F g^−1^, which considerably increased relative to chemically coprecipitated MnO_2_ (146.5 F g^−1^). The specific capacitance was reduced by just 6% following 600 cycles due to the high material cycling performance. Zefirov et al. [91] used an organometallic compound dissolved in supercritical carbon dioxide in an organometallic precursor to prepare MnO_2_ nanoparticles with small grains and a low polydispersity index.

In summary, the advantages of the microemulsion method had a simple experimental set-up and low energy consumption, enabled easy handling, and had potential for commercial production. However, the microemulsion process requires excessive solvent [92].

### 3.7. Chemical Coprecipitation

Chemical coprecipitation is used for synthesizing composites containing two or more metallic elements. In this method, nanoparticle precipitates can be generated through a controlled reaction of cations with anions. This reaction may be impacted by temperature, pH, and reactant concentration [93].

**Figure 6:**
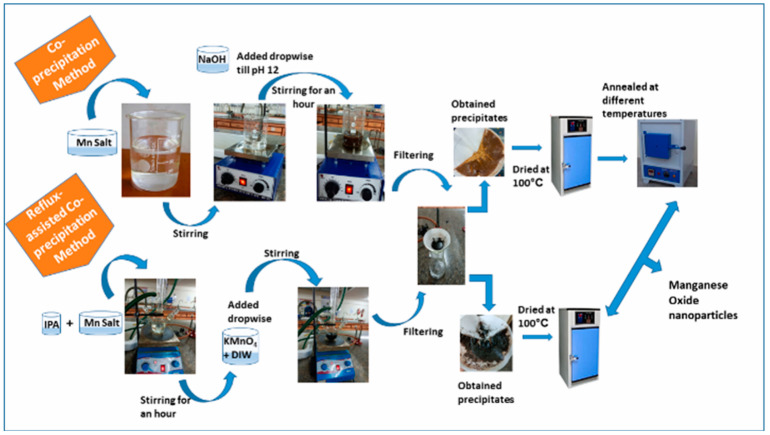
Schematic representation of MnO_2_ nanoparticle synthesis process [95].

Sivakumar et al. [94] synthesized α-MnO_2_ nanoparticles via chemical coprecipitation. The results of cyclic voltammetry analysis showed α-MnO_2_ nanoparticles had good capacitive behavior. Yaday et al. [95] synthesized MnO_2_ nanoparticles using simple chemical coprecipitation and reflux-assisted coprecipitation methods at different reflux durations and annealing temperatures separately. XRD, FTIR spectroscopy, UV-vis spectroscopy, BET surface area analyzer, and thermogravimetric analysis were utilized to examine sample optical, structural and thermal performances. The Scherrer equation was utilized to evaluate the mean sample grain size, which was determined to be 6~8 nm (6~7 nm) and 15~30 nm (20~46 nm) for reflux-assisted and coprecipitation approaches, separately. The peaks correspond to Mn-O bonds on the FTIR spectra, verifying that MnO_2_ nanoparticles were formed. According to FESEM analysis, the samples had nanorod-type morphology. MnO_2_ nanoparticles exhibited pseudo-capacitive behavior and excellent photocatalytic performance for the degradation of bright green dyes. Figure 6 displays the MnO_2_ nanoparticles preparation route. Pan et al. [96] prepared five crystalline forms of MnO_2_ with manganese sulfate being the manganese source and investigated differences in physicochemical properties based on specific surface area, phase morphology, pore volume, pore size, surface structure and particle size. The performance tests and electrode reaction kinetics for the five crystal batteries and capacitors showed that δ-MnO_2_ and γ-MnO_2_ are more suitable for capacitors and batteries, respectively.

The chemical co-precipitation method requires low reaction temperature and simple equipment and has low energy consumption, safe operation, simplicity, and low cost. However, chemical co-precipitation also has drawbacks: (1) the prepared manganese dioxide material is relatively low in purity and (2) poor homogeneity and being prone to agglomeration problems, which affects material properties.

### 3.8. Chemical Reduction Method

Chemical reduction has been developed as the high-efficiency wet chemical approach used to synthesize zero-valent nanoparticles. It is commonly used for synthesizing magnetic metal nanoparticles such as iron, cobalt, and nickel [97]. The average particle size and distribution can be controlled by adjusting the preparation conditions such as the concentrations of solvents, surfactants, and reducing agents [98].

Li et al. [99] provided an easy and high-efficiency solid-solution reaction pathway at a low temperature (60 °C) without using templates or surfactants for the large-scale synthesis of α-MnO_2_. α-MnO_2_ is a new candidate material for lithium battery applications. Khan et al. [100] prepared MnO_2_ nanoparticles and MnO_2_ nanoparticle/activated carbon (MnO_2_/AC) composites via chemical reduction. The results showed that the MnO_2_/AC composite contributed to degrading CR (Congo Red) dye by ~98.53%, whereas MnO_2_ nanoparticles degraded CR dye by 66.57% under the identical irradiation time. Moreover, the MnO_2_/AC composite was highly sustainable and could be used for repeated degradation of CR dye after rinsing and thermal treatment. Cremonezzi et al. [101] synthesized highly capacitive δ-MnO_2_ using a new easy route by reducing potassium permanganate. The capacitance of δ-MnO_2_ was 190 F g^−1^ at 0.25 A g^−1^.

In summary, the chemical reduction method for the preparation of MnO_2_ nanoparticles has advantages such as the low cost and ready availability of raw materials, straightforward operational procedures, and ease of control. However, the chemical reduction method has some limitations in the reducing agent such as high toxicity, low purity, and high synthesis costs.

### 3.9. Green Synthesis Method

Recently, more and more studies have been conducted to prepare manganese dioxide nanoparticles by the green synthesis methods. This phenomenon indicates that the greening of preparation methods will be a significant direction in the future. Green synthesis is an ecofriendly, cleaner, and cheaper method for nanoparticle synthesis. This method is viable for synthesizing biocompatible nanoparticles, thereby bridging materials science and biotechnology. Moreover, nanoparticles with controllable shapes and sizes can be prepared via green synthesis [102]. Fruits, vegetables, plant extracts, fungi and microorganisms have been used as raw materials to prepare manganese and manganese-oxide nanoparticles via green synthesis [103].

#### 3.9.1. Plant Extraction Method

The environmentally friendly preparation of MnO_2_ nanoparticles with plant extracts can be an economical and effective method [104]. In this method, plant extracts are added to a metal salt solution at room temperature, and this reaction can be completed within several minutes. Metal reduction can be achieved by diverse compounds including terpenoids, polysaccharides, phenolics and flavonoids in plant extracts [105]. At present, some plant extracts are adopted for synthesizing MnO_2_ nanoparticles.

**Figure 7:**
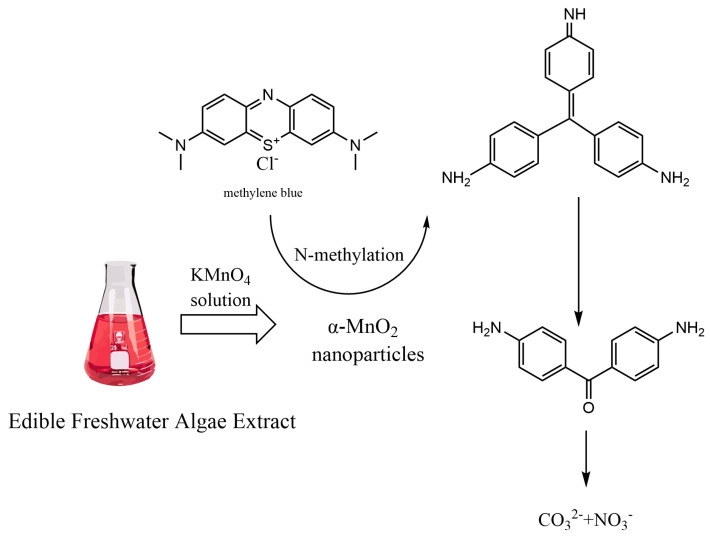
Degradation of toxic dyes by green synthetic manganese dioxide nanoparticles.

Hashem et al. [106] prepared MnO_2_ nanomaterials via the green synthesis of lemon peel (P) or juice (J). The crystalline and electrochemical properties of P-MnO_2_ and J-MnO_2_ were improved since lemon peel possesses 3 reducing reagents, and lemon juice contains citric acid and ascorbic acid. P-MnO_2_ have the same electrochemical properties as conventional reducing reagents, but P-MnO_2_ was expensive. The novel preparation method is simple, cost-effective, environmentally friendly, and scalable for large-scale α-MnO_2_ nanoparticle synthesis. The MnO_2_ nanoparticles can be applied to electrochemical energy storage. Shehroz et al. [107] prepared the three MnO_2_ three phases (α-, β-, and γ-MnO_2_) in a single individual. For this purpose, natural surfactants were synthesized using bitter apple extract as a green solvent. MnO_2_ nanoparticles were synthesized under the same conditions with/without plant extracts. Experimental results showed that the average size of products was 20~50 nm by the green synthesis method, while that was 20~25 nm for nanoparticles prepared by chemical methods. Dye and nitroaromatic reduction was investigated by using MnO_2_ nanoparticles as the catalysts. Moreover, the apparent rate constants, reduction rates, reduction concentrations, and reduction time were analyzed. The nanoparticles prepared by the environmentally friendly method showed superior catalytic performance to those prepared by the chemical method. Ramesh et al. [108] synthesized green MnO_2_ nanoparticles using medicinal plant extracts. The results of XRD analysis proved the crystal structure of MnO_2_ nanoparticles. The results of SEM illustrated that MnO_2_ nanoparticles prepared by the environmentally friendly method showed a spherical shape. Moreover, 72% of methylene blue (MB) dye was degraded after 150 min under UV light irradiation. Table 6 summarizes the applications of MnO_2_ synthesized using the plant extraction method, the prepared MnO_2_ nanoparticles have different sizes with the different plant sources. MnO_2_ has good applications in heavy metal adsorption, organic pollutant adsorption and so on. The degradation mechanism of toxic dyes by green synthesized manganese dioxide nanoparticles is shown in Figure 7.

In summary, the yield of MnO_2_ nanoparticles prepared by the plant extraction method is low compared with those prepared by other methods, and it can hardly control the generation conditions of nanoparticles precisely, and the products prepared by the plant extraction method still have certain toxins [119]. In contrast to other preparation methods, the plant extraction method possesses the advantages of being cleaner and more conducive to sustainable development.

#### 3.9.2. Environmentally Friendly Synthetic Methods Based on Microorganisms

Microorganisms are promising for nanoparticle synthesis. Metal salts are reduced into metal nanoparticles via the domestication of enzymes. Fungi exhibit superior bioaccumulation and resistance, which contribute to synthesizing metal nanoparticles. The interaction of microorganisms with metals is also extensively investigated [120]. Microorganisms can be adopted for accumulating or extracting metals via bioleaching, bioremediation, and heavy metal elimination [121].

Sinha et al. [122] synthesized intracellular MnO_2_ nanoparticles by the simultaneous manganese remediation from the highly mono-disperse medium using Bacillus sphaericus. Those prepared nanoparticles were orthorhombic crystalline MnO_2_. When cells were challenged with manganese, MnO_2_ nanoparticles (mean size, 4.62 ± 0.14 nm) were prepared. The above study offered the merits of synthesizing relevant oxide nanoparticles to prevent manganese pollution. Borah et al. [123] achieved a compositionally controllable, room-temperature, and simple environmentally friendly preparation route of high-purity α-MnO_2_ nanoparticles by reducing KMnO_4_ aqueous solution with an edible freshwater red algae aqueous extract. The synthesized MnO_2_ nanoparticles showed excellent photocatalytic performance for rhodamine B (RhB), methylene blue (MB) and methyl Orange (MO), with degradation rate constants of 0.06781, 0.03831 and 0.04323 min^−1^, separately. The photocatalysts were easily recycled and highly stable. In total, 3 mg of MnO_2_ nanoparticles exhibited nearly total degradation efficiency (92%) within 30 min. Alvares et al. [124] used *Haloarchaea alexandrina* GUSF-1 cell lysates to obtain Mn_3_O_4_-MnO_2_ nanocomposites. The antimicrobial activity of these nanocomposites satisfied *Pseudomonas aeruginosa* > *Salmonella typhimurium* > *Escherichia coli* > *Amoebacterium* commonly known as *Proteus mirabilis* > *Candida albicans* > *Staphylococcus aureus*.

In summary, the environmentally friendly synthetic methods based on microorganisms exhibit the advantages of being environmentally friendly and possessing good biocompatibility. However, the synthesis method is easily influenced by different factors [125,126], like strain type and environmental conditions such as temperature, pH, salt concentration and growth medium, all of which have direct or indirect influence on nanoparticle composition, size and morphology. As a result, it also faces challenges such as high technical difficulty, low stability, issues with purity, and concerns over biosafety.

## 4. Electrochemical Applications

MnO_2_ nanomaterials can be used in supercapacitors and batteries due to them being inexpensive, widely available, and malleable [127]. Table 7 summarizes other electrochemical applications of manganese dioxide, which can be used in zinc-ion batteries, pneumatic actuators, and so on.

### 4.1. Supercapacitors

Supercapacitors are energy-storage technologies widely researched recently. Unlike batteries, supercapacitors can be rapidly recharged, operate at a wider temperature range, are environmentally friendly, and offer better safety, higher reliability, and maintenance-free operation [132]. Moreover, the electrochemical properties are largely determined by active substances contained within the electrodes. MnO_2_ nanomaterials can be used to prepare high-performance electrode materials for supercapacitors because of their large specific capacity and good electrochemical performance. MnO_2_ materials can store and release electrical energy quickly and display excellent cycle stability. Additionally, the energy-storage properties of supercapacitors are further enhanced by controlling MnO_2_ material characteristics, such as morphology, crystal structure, and pore structure. Supercapacitors can be used in printed electronics [133], electric vehicles [134], smart devices [135], and energy-storage systems [136].

Conventional supercapacitors use activated carbon-based materials as electrodes. This material had typical carbon-based material advantages, including abundant material sources, environmentally friendly properties, excellent electroconductivity, high specific surface area, and broad operating temperatures [137]. Electrochemical capacitors containing carbon-based materials were electrochemical double-layer capacitor types. The capacitance depends on the accessible electrolyte ion surface area rather than the capacitor material body. The carbon-based materials provide a high specific surface area, their pore size distribution and pore structure affect the energy storage rate of EDLC supercapacitors [138].

Electrochemical double-layer capacitors use materials with limited capacitance and supercapacitor materials with pseudo-capacitance may be 10~100 times more capacitive. The store charge is similar to conventional capacitor electrodes and exhibits a Faraday reaction between the electrode material and ions. Such pseudo-capacitive supercapacitor materials are divided into two types: excessive metal oxides or conducting polymers [139,140,141]. Excessive metal oxides include ruthenium oxides, manganese oxides, and nickel oxides [142,143,144]. To be specific, metal oxides offer increased energy density compared with traditional carbon-based materials. The pseudo-capacitance of metal oxides is affected by physical properties and chemical factors [145,146]; however, they can yield higher performances by modifying or using composite materials as well as the adjustment of electrode structure.

Yao et al. [147] obtained an excellent capacitance of MnO_2_ electrode material by printing pseudo-capacitor electrodes. The MnO_2_ electrode was loaded with 182.2 mg cm^−2^ and its capacitance was 44.13 F cm^−2^. The specific capacitance of the 2D MnO_2_/pSiNW electrode prepared by Bagal et al. [148] was 311.89 F g^−1^ at 2 A g^−1^. Using it as the anode, the density and power density of this capacitor were the highest (93.31 mWh cm^−2^ and 1.51 mW cm^−2^, separately), while its capacitance retention was 89.5% over 10,000 cycles. Tynan et al. [149] uniformly deposited MnO_2_ nanoparticles with pseudo-capacitance on carbon nanotubes using the chemical method, and the capacitance of MnO_2_ nanoparticle electrodes could be enhanced by a factor of 9 relative to the benchmark material at a loading of 95 wt% of MnO_2_. Moreover, MnO_2_ nanoparticles enhanced the structure of hybrid electrodes, such as a 110% and 430% increase in tensile strength and stiffness compared to the benchmark material. Table 8 summarizes the different synthesis methods and forms of MnO_2_ used as supercapacitors. It elucidates the specific capacitance, energy density, scan rate, and cycling stability, with the results indicating that the manganese dioxide prepared via the hydrothermal method and doped with Ag exhibits a maximum specific capacitance of 1027 F g^−1^, at a scan rate of 1 A g^−1^. Although the two-dimensional layered δ-MnO_2_ prepared by the chemical reduction method possesses a relatively low energy density, it exhibits exceptional cycling stability, retaining 98.7% of its initial performance after 10,000 cycles. In contrast, the cycling stability of manganese dioxide prepared by electrodeposition is relatively poor, achieving only 56.81% after 1000 cycles, as compared to other methods. δ-MnO_2_ materials successfully prepared by a chemical reduction method, and thoroughly evaluated the electrochemical properties of these materials, as well as their composites with carbon (C, labeled as C/MnO_2_ with varying reaction times of 0.5 h, 1 h, and 2 h), using cyclic voltammetry (CV) and galvanostatic charge–discharge (GCD) tests in a standard three-electrode system with 1.0 M sodium sulfate electrolyte. At a scan rate of 200 mV/s, the CV curve of pure carbon (C) exhibited a near-rectangular shape, clearly indicating its excellent electric double-layer capacitance behavior during both anodic and cathodic scans. Similarly, the CV curves of the C/MnO_2_ nanocomposites also displayed a quasi-rectangular shape with no pronounced redox peaks, revealing a synergistic effect between the electric double-layer capacitance and the rapid, reversible Faradaic redox reactions occurring on the MnO_2_ surface, operating at a pseudo-constant rate across the entire potential range. Notably, the C/MnO_2_ sample prepared for 1 h exhibited the largest CV area, signifying its possession of the highest specific capacitance. During the charge–discharge tests, the GCD curves of all samples maintained an almost perfect triangular shape, which not only attested to the materials’ extended charge–discharge durations but also highlighted the substantial positive contribution of pseudocapacitive mechanisms to the overall specific capacitance. Across a wide range of current densities from 0.25 to 10 A g^−1^, the GCD curves of all samples remained close to triangular, demonstrating ideal capacitive behavior and high Coulombic efficiency. It is noteworthy that while the pure carbon material (C) displayed good rate capability, its specific capacitance fell below 50 F g^−1^. In contrast, the C/MnO_2_ sample prepared for 1 h achieved the highest specific capacitance of 116.61 F g^−1^ at a current density of 1 A g^−1^, significantly surpassing that of C/MnO_2_ prepared for 0.5 h (84.65 F g^−1^) and 2 h (58.37 F g^−1^), likely due to the optimized nanosheet structure and appropriate composition. It was also observed that as the current density increased, the specific capacitance of all electrode materials decreased gradually. This phenomenon can be attributed to the fact that electrolyte ions can diffuse sufficiently and uniformly into the internal pores of the electrode materials at low current densities, enabling a higher specific capacitance. However, the electrolyte ions are time-constrained and fail to adequately access all active sites within the electrode under the high current densities, leading to insufficient Faradaic redox reactions and, consequently, a lower specific capacitance.

In summary, supercapacitors can be used in many applications, but their performance is considerably affected by electrode materials. MnO_2_ and its composites can improve cycle life, power density, and energy density compared with traditional carbon-based materials and may be potentially applied in large-scale energy storage.

### 4.2. Zn-MnO_2_ Batteries

Zn-MnO_2_ batteries are a common type of disposable batteries and typically comprise Zn and MnO_2_ as anode and cathode, separately, and an electrolyte. Compared with other batteries, Zn-MnO_2_ batteries are inexpensive, have better stability and longer storage life, and are environmentally friendly and recyclable [162]. Zn-MnO_2_ batteries are mainly used in electronic devices [163].

In neutral and weakly acidic electrolytes, MnO_2_ in Zn-MnO_2_ batteries are first reduced to MnOOH. As the acidic solubility increases, MnOOH is reduced to Mn^2+^ and Zn metal is oxidized to Zn ions. This redox reaction generates an electric current in the battery, thus realizing electrical energy conversion and storage. The Zn-MnO_2_ battery performance is affected by physical conditions and chemical factors [164,165]. However, the performance is enhanced after adding electrolytes. Shen et al. [166] found that redox conversion of MnO_2_ with Mn^2+^ could be achieved by maintaining critical range conditions. Zn-MnO_2_ batteries based on this electrochemical property can withstand 16,000 cycles without significant capacity degradation, and the stored energy density was 602 Wh kg^−1^. Liu et al. [167] modulated the electrolyte composition by adding acetic acid and chromium chloride (Cr^3+^) and using combined strategies such as pre-cycling and sonication. MnO_2_ suspension was mitigated, and a more stable and reversible cycling reaction was achieved after combining pre-cycling and sonication. The modified zinc-MnO_2_ batteries showed higher Coulombic efficiency at 1.4 V and maintained 7500 stable cycles, and the capacity and current density were 0.5 mAh cm^−2^ and 10 mA cm^−2^ separately. Ma et al. [168] added an aqueous organic electrolyte of tetra-ethylene glycol dimethyl ether to inhibit water molecule activity, thus avoiding the generation of by-products. The specific capacity of Zn-MnO_2_ batteries was as high as 132 mAh g^−1^. The capacity retention reached >98% following 1000 cycles at the 1.25 V operating voltage and the 200 mA g^−1^ current density. Each of these studies demonstrates that Zn-MnO_2_ cell performance may be improved by adjusting the electrolyte composition and employing specific strategies. Table 9 summarizes the comparison of different crystals of manganese dioxide in zinc–manganese batteries. The results indicate that δ-MnO_2-x_ exhibits higher capacitance compared to several other electrode materials, primarily due to the importance of layered structure in enhancing capacitive performance. This unique structure favorably facilitates the surface adsorption and intercalation of metal cations such as Na^+^, K^+^, and H^+^. Consequently, it enables the reversible transition between Mn (IV) and Mn (III) valence states, which is vital for charge storage. Essentially, the layered structure of δ-MnO_2_ promotes efficient ion transport and electron transfer, thereby elevating its overall capacitive performance. However, β-MnO_2_ electrodes prepared via the electrodeposition method exhibit the longest cycle life. This is attributed to the ability of the electrodeposition process to precisely control the thickness and structure of the β-MnO_2_ deposit, resulting in a uniform and dense layer. Additionally, the tunnel structure and chemical stability of β-MnO_2_ facilitate rapid ion transport and charge storage, while minimizing material degradation during cycling. Consequently, β-MnO_2_ electrodes produced through the electrodeposition method are able to demonstrate extended cycle life. The electrochemical properties of β-MnO_2_ material were comprehensively evaluated using cyclic voltammetry (CV). Within the discharge potential range of approximately 1.8–2 V, paired with the Zn^2+^/Zn system, the material exhibited stable areal capacity performance over the initial 20 cycle periods. Notably, under a 2.2 V charging condition, a uniform layer of manganese dioxide was observed to cover the C-cloth CC substrate, clearly indicating the achievement of homogeneous and dense deposition of manganese dioxide nanoflowers on the C-cloth surface. This phenomenon was robustly supported by the stability of the discharge platform over the first 20 cycles, further attesting to the remarkable thermodynamic stability of β-MnO_2_ within the Zn–manganese dioxide battery system and its ability to maintain a more regular morphological structure.

In general, Zn-MnO_2_ batteries, as a kind of low-cost battery, offer a reasonable capacity and energy density. Therefore, Zn-MnO_2_ is widely adopted for electronic devices, and its performance, although affected by many factors, can still be improved by optimizing the electrolyte composition. Zn-MnO_2_ batteries have a broad application prospect in the energy-storage field

### 4.3. MnO_2_/Carbon Nanomaterial Composites

MnO_2_/carbon nanomaterials composites (MnO_2_/CNTs) are nanocomposites integrated with the unique properties of MnO_2_ and carbon nanotubes. Since carbon nanotubes possess superb mechanical stability, increased surface area, and great electrical conductivity, the integration of CNTs with manganese dioxide efficiently enhances the specific capacity, conductivity, as well as other electrochemical properties of the composite [175]. In addition, CNTs contribute to improving cycle stability and charge/discharge rate capability of the MnO_2_/CNTs composites [176]. Therefore, MnO_2_/CNT composites exhibit superior electrochemical properties. MnO_2_/CNTs are mainly used in supercapacitors [177], biosensors [178], catalysts [179] and other applications.

Li et al. [180] successfully recovered MnO_2_/CNT cathodes from MnO_2_ electrodes by simple calcination at mild temperatures and used then to be electrodes in supercapacitors. The specific capacity was 253.86 F g^−1^ within the 0.5 M Na_2_SO_4_ at 0.5 A g^−1^. The sustainability of carbon-based materials for high-performance electrochemical applications was demonstrated through recycling. Rosaiah et al. [181] prepared and investigated electrochemical performances of pure manganese dioxide and MnO_2_/CNTs composites by a hydrothermal synthesis method, and discharge capacities were 1225 and 1589 mAh g^−1^, separately. The MnO_2_/CNTs composites exhibited high stability, and the capacitance was 957 mAh g^−1^ following 60 cycles. The superb specific capacity and cycling performance are associated with the synergistic effect of carbon matrix materials with MnO_2_, and this synergistic effect also indicates that carbon matrix materials are important for MnO_2_/CNT composites. Zhou et al. [182] prepared MnO_2_@CNTs composite electrodes that possessed the 3D nanostructure. The capacitance of the prepared MnO_2_@CNTs composite electrodes reached 256 mAh g^−1^ at 0.1 A g^−1^ and remained stable following 700 cycles. This work explains the mechanisms by which carbon nanotubes enhance MnO_2_ cathode performances, providing a new perspective on designing efficient electrochemical energy storage devices.

To sum up, the carbon matrix material plays a central role and significantly improves the composite electrochemical performances in the MnO_2_/CNTs composites. Meanwhile, the sustainable characteristics of carbon matrix materials contribute to their broad uses in energy storage.

## 5. Summary and Outlook

**Figure 8:**
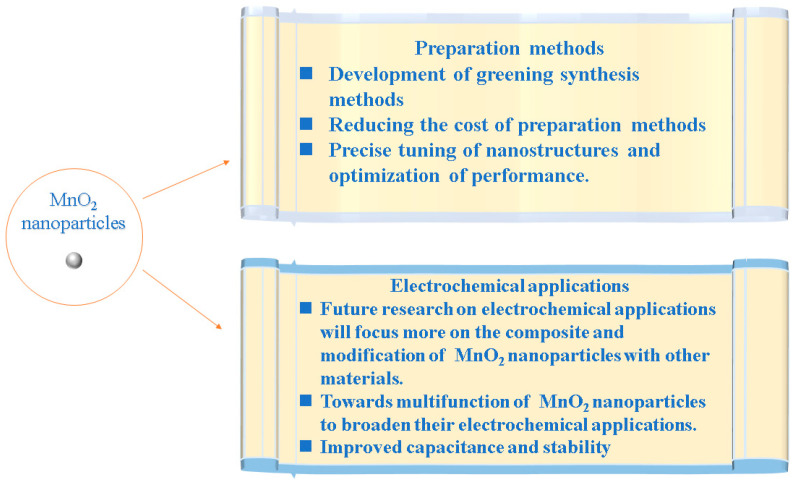
Future aspects of MnO_2_ nanoparticles.

In conclusion, MnO_2_ is being widely used due to its unique properties. In contrast to the previous review, this review summarizes approaches for preparing MnO_2_ nanoparticles and describes the corresponding respective merits, demerits or limitations, which is believed to help researchers to better select the synthesis methods. In addition, the multifunctional extension applications of MnO_2_ nanomaterials are also presented. Although great progress is achieved in MnO_2_ nanoparticle studies, the preparation methods and the electrochemical applications of MnO_2_ nanoparticles need to be further investigated. Here, the future research prospects of MnO_2_ nanoparticles are briefly discussed, as shown in Figure 8.

Up to now, the preparation method of MnO_2_ nanoparticles has been continuously improved, and the performance of MnO_2_ nanoparticles has been greatly improved. However, the research on MnO_2_ nanoparticles is still in its infancy and has not been fully applied to practical applications. Addressing this challenge will require focused efforts in several areas in the future.

i. Although there are several ways to prepare MnO_2_ nanoparticles, realizing large-scale, cost-effective and high-quality synthesis remains challenging. The high production cost makes the commercialization of manganese dioxide nanoparticles difficult, especially in cost-sensitive industries.
ii. Although MnO_2_ nanoparticles have demonstrated excellent performance on the laboratory scale, a series of technical challenges need to be addressed in practical applications, for example, how to improve the stability and electrochemical properties of MnO_2_ nanoparticles. These issues need to be addressed by continuous research and technological innovations.
iii. Despite the excellent MnO_2_ nanoparticle performances, the process of MnO_2_ preparation may generate some hazardous substances and wastes. This requires manufacturers to take environmental protection measures during the production process. In addition, the environmental impacts of nanomaterials need to be further studied and evaluated.

## Figures and Tables

**Table 1:** Structures of MnO_2_ materials.

Crystalline Morphology	Structure Type	Tunnels (n × m)	Dimension	Reference
α-MnO_2_	Hollandite	(2 × 2)	1D	[14]
β-MnO_2_	Pyrolusite	(1 × 1)	1D	[15]
γ-MnO_2_	Nsutite	(1 × 1)/(1 × 2)	1D	[16]
δ-MnO_2_	Birnessite	(1 × ∞)	2D	[17]
λ-MnO_2_	Spinel	(1 × 1)	3D	[18]
ε-MnO_2_	-	(1 × 1)/(1 × 2)	3D	[19]

**Table 4:** Structure and application of MnO_2_ prepared using the hard template method.

Preparation Products	Formwork	Experimental Data	Applications	Reference
MnO_2_@polypyrrole	Polystyrene	The specific capacitance, energy density, and power density were 63 F g^−1^, 42 Wh kg^−1^ and 1100 W kg^−1^, separately.	Supercapacitors	[60]
S/MnO_2_-280H	S	The capacitances of 1053 and 551 mAh g^−1^ following 400 cycles	Cathodes with Li-S batteries	[61]
MnO_2_ (KIT-6)	KIT-6	The bifunctional activity measurable value of 1.28 V	Electrocatalysts	[62]
Flower-like MnO_2_	MnCO_3_ microspheres	90% removal of 1000-ppm toluene	Catalyst	[63]

**Table 5:** Structure and application of MnO_2_ prepared by the electrodeposition method.

Synthetic Structure	Measurement Conditions	Performance	Applications	Reference
MnO_2_/poly (3,4-ethylenediox-ythiophene) (PEDOT)	10 mV s^−1^	Capacitance was 89.7 mF cm^−2^	Supercapacitors	[74]
γ-MnO_2_	0.025 V s^−1^	The capacitance was 43.1 F g^−1^	Capacitor electrodes	[75]
ε-MnO_2_	100 mAh g^−1^	The discharge capacity delivered by the cell was 5700 mAh g^−1^	Li-O_2_ Catalysts	[76]
MnO_2_ nanostructures	1 A g^−1^	Capacitance and stability were 369 F g^−1^ and 97% following 1000 cycles	Supercapacitors	[77]
MnO_2_ nanowires	1 mA cm^−2^	The stability was 92.6% after 10,000 cycles	Supercapacitors	[78]
MnO_2_@Mn	0.86 V	The catalyst showed good stability after a 30h timed current test with little or no decay	Catalysts	[79]
MnO_2_-NiFe/Ni	50 mA cm^−2^	The power density was 93.95 mW cm^−2^	Oxygen electrocatalysts	[80]
α-MnO_2_/γ-MnO_2_	193 µW cm^−2^	The energy density was 93.8 µWh cm^−2^	Supercapacitors	[81]

**Table 6:** Preparation of MnO_2_ nanoparticles with plant extracts and their applications.

Plant Organism	Nanoparticle Structures of MnO_2_	Particle Size	Effect	Appliance	Reference
Flower extract	MnO_2_ nanorods	100 nm	Decolorization of the target dye was 91.3%. TOC and COD were reduced by 90.6% and 92.1% separately.	Removal of crystalline violet dye	[109]
Saraca asoca leaves extract	MnO_2_ nanoparticles	18 nm	The semi-inhibitory concentration values of 20 µg/mL for both MCF-7 and MDAMB-231 cells	Considerable cytotoxic effects on cancer cells	[110]
Yucca gloriosa leaf extract	MnO_2_ nanoparticle	80 nm	The photocatalytic efficiency for 20 min was 33%	Photocatalytic activity and good degradation of organic dyes	[111]
Potato leaf extract	MnO_2_ nanoparticle	26 nm	Significant increases of 67.1% in plant growth activity, 52.8% in photosynthetic pigments, and 56.25% in non-enzymatic antioxidant activity in soil, respectively	Multi-aspect enhancer	[112]
Extract of viola betonicifolia	Green synthesized MnO_2_ nanoparticles and Chemically Synthesized MnO_2_ Nanoparticles	10.5 ± 0.85 nm	Cell survival (79.33 ± 0.75%), (73.54 ± 0.82%), respectively	Used to provide antimicrobial coatings	[113]
Extract of ficus retusa plant	α-MnO_2_ nanoparticles	30~50 nm	The adsorption capacities for Mo and Mr dyes were 116.1 and 74.02 mg g^−1^, separately	Adsorbent	[114]
Papaya leaf extract	MnO_2_ nano-conjugate	30~40 nm	The urea and cholesterol reduced to 94 ± 2.16	For the treatment of hyperbilirubinaemia	[115]
Chamomile flower extract	MnO_2_ nanoparticles	16.5 nm	The percentage of apoptotic cells in RS-2 ranged from 0.97% to 99.94%	Strong inhibitory effect on rice strain RS-2	[116]
Plant extracts	α-MnO_2_	2.8~4.5 nm	The capacitance and stability were 500 F g^−1^ and 71%, separately, after 7000 cycles	Supercapacitors	[117]
Mango lead extract	δ-MnO_2_ nanoparticles	1.5~2.5 nm	The efficiency with >96% removal of cationic pollutants	Cation adsorbent	[118]

**Table 7:** Electrochemical applications of MnO_2_.

Synthetic Structure	Measurement Conditions	Performance	Applications	Reference
β-MnO_2_/Polypyrrole	0.2 A g^−1^	Specific discharge capacity of 361.7 mAh g^−1^	Zinc-ion batteries	[128]
Manganese dioxide/gelatin-glycerol	±2 V	High bending actuation (20-mm deflection, >360° scan angle, and 2.5-mm radius of curvature) and different shape change	Air-working actuator	[129]
α-MnO_2_	0.1 A g ^−1^	Capacity was 190 mAh g^−1^ and the stability was after 50,000 cycles in (NH_4_)_2_SO_4_	Ammonium-ion energy storage	[130]
MnO_2_/graphitic carbon nitride (g-CN)	5 mV/s	The optimal composite system achieved a current density of 10 mA/cm^2^ with an overpotential of 430 mV and exhibited a Tafel slope of approximately 70 mV/dec	Electrocatalysts	[131]

**Table 8:** Comparison of energy storage performance of different synthesized and formed manganese dioxide in supercapacitors.

Material	Preparation Method	Specific Capacitance	Cycling Life	Energy Density	Reference
α-MnO_2_	Plant extraction method	90 F g^−1^ at 1 A g^−1^	98% after 1000 cycles	37 Wh kg^−1^	[150]
δ-MnO_2_	Chemical reduction method	116.61 F g^−1^ at 1 A g^−1^	98.7% after 10,000 cycles	22.7 Wh kg^−1^	[151]
MnO_2_/Ag	Chemical reduction method	115 F g^−1^ at 0.2 A g^−1^	75% after 1000 cycles	45 Wh kg^−1^	[152]
MnO_2_-NiO	Electrodeposition method	375 F g^−1^ at 0.5 A g^−1^	56.81% after 1000 cycles	-	[153]
Ag_0.05_ MnO_2_	Hydrothermal method	1027 F g^−1^ at 1 A g^−1^	93.16% after 10,000 cycles	-	[154]
ZnO@MnO_2_	Hydrothermal method	839.9 F g^−1^ at 0.3 A g^−1^	92% after 10,000 cycles	74.6 Wh kg^−1^	[155]
α-MnO_2_	Hydrothermal method	47 F g^−1^ at 0.5 A g^−1^	94% after 5000 cycles	21 Wh kg^−1^	[156]
λ-MnO_2_/polyaniline	Hydrothermal method	232.1 F g^−1^ at 0.2 A g^−1^	78.65% after 3000 cycles	66.4 Wh kg^−1^	[157]
β-MnO_2_	Hydrothermal method	212.85 F g^−1^ at 0.2 A g^−1^	97.5% after 5000 cycles	-	[158]
γ-MnO_2_	Hydrothermal method	103 F g^−1^ at 1 A g^−1^	-	-	[159]
Polyaniline-MnO_2_	Templates method	765 F g^−1^ at 0.25 A g^−1^	80% after 14,000 cycles	-	[160]
Polyaniline-MnO_2_	chemical co-precipitation method	417 F g^−1^ at 5 mV s ^−1^	-	7.2 Wh kg^−1^	[161]

**Table 9:** Comparison of different crystalline manganese dioxide in zinc–manganese batteries.

Cathode	Preparation Method	Electrolyte	Plateau (V)	Capacity (mAh g^−1^)	Cycling Life	Reference
α-MnO_2_	Hydrothermal method	2 M ZnSO_4_ + 0.1 M MnSO_4_	0.8–2.0	302	78.4% after 2000 cycles	[169]
β-MnO_2_	Hydrothermal method	2 M ZnSO_4_ + 0.1 M MnSO_4_ + 0.1 M Na_2_SO_4_	1.0–1.9	325	94% after 1000 cycles	[170]
δ-MnO_2-x_	Hydrothermal method	2 M ZnSO_4_ + 0.1 M MnSO_4_	0.9–1.9	551.8	83% after 1500 cycles	[171]
ε-MnO_2_	Hydrothermal method	3 M MnSO_4_ + 0.3 M H_2_SO_4_ + 0.06 M NiSO_4_	1.16–3.4	270	99% after 450 cycles	[172]
β-MnO_2_	Electrodeposition method	1 M ZnSO_4_ + 1 M MnSO_4_	1.8–2.2	-	≈100% after 400 cycles	[173]
γ-MnO_2_	Electrodeposition method	0.5 M Mn (CH_3_COO)_2_ + 0.5 M Na_2_SO_4_	-	391.2	92.17% after 3000 cycles	[174]
